# Neural responses to light stimulation in the octopus arm

**DOI:** 10.1242/jeb.250111

**Published:** 2025-03-31

**Authors:** Weipang Chang, Melina E. Hale

**Affiliations:** Organismal Biology and Anatomy, University of Chicago, Chicago, IL 60637, USA

**Keywords:** Cephalopod, Dermal light sense, Sensory system

## Abstract

Octopuses are known to be visual animals. Beyond functions of the eyes, recent investigations have documented the importance of extraocular photoreception in behavior. Octopus arms have been shown to respond behaviorally to local light exposure with negative phototaxis. Moreover, light-activated chromatophore expansion (LACE) in octopus arms indicates that skin-based photoreception may contribute to light detection. In this study, we used electrophysiological recordings to investigate the neural activity of the arm's axial nerve cord in response to light on the arm. We tested the hypothesis that light stimulates the activity of neurons in the arm's axial nerve cord. We also aimed to determine sensitivity to different wavelengths of light. The results showed that the axial nerve cord is strongly responsive to light stimulation of the arm and that the response travels along the length of the axial nerve cord. Blue light generated the strongest neural activity while red and green light also induced responses. Light-induced neural activity was mediated through the aboral arm skin and by the oral-side skin and suckers. These findings reveal the role of the skin in the sensory abilities of octopuses and provide insights into the neural mechanisms underlying their response to light. Our study underscores the importance of extraocular photoreception in future investigations of cephalopod sensory and behavioral biology.

## Teaser

**Highlighted Article:** The axial nerve cord of the octopus arm responds physiologically to local light stimulation and particularly strongly to blue light; responses are mediated through the arm skin, highlighting its role in light detection.

## INTRODUCTION

Octopuses are visual animals and there is increasing appreciation that their light reception is not just eye based but also occurs through widespread extraocular photoreception. Early studies on denervated octopus and squid skin revealed that chromatophores in these cephalopods expand in response to light (Florey, 1966), suggesting an intrinsic sensitivity to light within their skin. More recent molecular analyses have identified transcripts for rhodopsin and retinochrome, critical components of light detection, in the skin and retina of squid and cuttlefish (Mäthger et al., 2010). Additionally, molecules associated with light detection have been found in chromatophores (Kingston et al., 2015; Ramirez and Oakley, 2015), and Ov-GRK1, a light-sensing molecule identified in the common octopus (*Octopus vulgaris*), was found distributed across various tissues, including suckers and skin (Al-Soudy et al., 2021). Behavioral studies further support roles for extraocular photoreception. Recently, light on the arm of freely behaving octopuses has been shown to elicit a negative phototactic response (Katz et al., 2021). In addition, variation in the latency of light-activated chromatophore expansion (LACE) with stimulating wavelength has been recorded in *Octopus bimaculoides*; chromatophores exhibit a shorter latency to expand and are more responsive to blue light (470–480 nm) than they are to other wavelengths (Ramirez and Oakley, 2015).

While extraocular responses to light have been observed behaviorally and molecular mechanisms for light detection examined, the neural activity resulting from extraocular photoreception is unknown. Integration of extraocular photoreception into central sensory processing, potentially informing behavior, would change how we think about sensory processing and response coordination in these animals. Here, we examined the neurophysiological response to extraocular photoreception in the arms of young *O. bimaculoides*. We addressed the hypothesis that extraocular mechanisms for photoreception generate activity in the axial nerve cords of the arms, the main site of local arm neural circuits, and, in particular, transmission of signals from the arms to the brain. Although octopus arms respond to local stimulation without brain input (Boycott and Young, 1950), signaling with the brain is needed for most coordinated behavior. By examining activity in response to local light sensation in the cerebrobrachial tracts of the arm's axial nerve cord, we determined whether extraocular sensory input moves beyond local circuits and potentially to the brain and thus may participate in central decisions about behavioral responses.

Based on previous research demonstrating that chromatophores are responsive to various wavelengths of light, with a pronounced sensitivity to blue light (Ramirez and Oakley, 2015), we hypothesized that the strength of neural responses in the axial nerve cord would vary depending on the wavelength of light applied to the skin, anticipating a stronger response to blue light than to red. To test this hypothesis, we recorded responses to stimulation by different wavelengths of light. Octopuses are understood to be color blind and differences in response with changes in light wavelength, as found by Ramirez and Oakley (2015), are intriguing. We conducted spike sorting analysis to evaluate whether neural responses recorded in the axial nerve cord varied with wavelength.

## MATERIALS AND METHODS

### Experimental animals

During all animal procedures, the regulations for cephalopod research worldwide, as outlined in EU Directive 63/2010/EU, were followed (Fiorito et al., 2015). Work was performed on young octopuses, *Octopus bimaculoides* Pickford & McConnaughey 1949 (2–4 months post-hatching; *N*=23, average mantle length 8.1±0.16 mm), that were obtained from the Marine Biological Laboratory, Woods Hole, MA, USA. Animals were kept individually in artificial seawater (ASW; Instant Ocean, S.G. 1.023; 16.53 g Instant Ocean per liter of water; salinity 1.020–1.025 ppt, pH 8.1–8.2) and standard recirculating water filtration. Water temperature was maintained at approximately 25°C and there was natural lighting. The ambient luminance during the day was around 300 lx. Octopuses were fed daily with pieces of shrimp and housing was cleaned daily. All efforts were made to utilize only the minimum number of experimental animals necessary to obtain reliable scientific data.

### Experiment overview

In order to examine neural responses to extraocular light sensation, we developed a physiology preparation of an isolated arm, removing any chance of input from the eyes. We recorded electrophysiologically from the axial nerve cord of the arm while varying the characteristics of the light stimulus and varying the region of skin and suckers stimulated.

### Dissection

Octopuses were euthanized using 330 mmol l^−1^ MgCl_2_ in ASW (Butler-Struben et al., 2018) and kept in the solution for 10 min until there was no response to pinch stimulation. After the cessation of any response to pinch stimulation, the head and much of the body were removed by cutting just below the eye level under the stereomicroscope (Leica MZFLIII, Leica Microsystems) and the arms and connected tissue were returned to ASW. For recordings on individual arms, arms were excised from the octopus and pinned to the recording chamber coated with Sylgard (Sylgard 184, Dow Corning Corporation). Tissues dorsal and lateral to the axial nerve cord were removed, exposing the cord for recording. For a subset of experiments that addressed hypotheses of regional differences in sensitivity and transmission of signals along the arm, we removed additional, specific areas of skin or suckers as specified in Results. In the process of octopus skin removal, the skin is carefully detached using fine tweezers, while the suckers are removed with fine scissors.

### Electrophysiology recording

Axial nerve cords of left side position 1 (L1) and 2 (L2) arms and of right side position 1 (R1) and 2 (R2) arms were selected for recording. The tissue was placed onto the recording chamber and the ASW was changed frequently for 20 min before the recording began. ASW (Instant Ocean) used for housing the octopus was also used for recording. Electrophysiological responses to light stimulation were recorded from the cerebrobrachial tract on the dorsal side of the axial nerve cord. The recording chamber was placed on a custom-built inverted microscope positioned on an upright compound base (Olympus) with a motorized *XY* stage (Prior Scientific). The optical fiber for light stimulation and recording electrodes were positioned by manipulators (Scientifica Inc.). Air-bubbled ASW was changed regularly throughout the duration of physiology experiments. The diameter of the axial nerve cord of a 2 month old octopus is less than 200 μm. We used suction electrodes with a relatively small tip diameter, 20 μm, to suck a portion of the axial nerve to record multi-unit responses to light stimulation. The electrodes were made by pulling borosilicate glass capillaries (GC150F-7.5 1.5 mm o.d., 0.86 i.d., Harvard Apparatus) in a P-97 Flaming/Brown micropipette puller (Sutter Instrument Co.). An Ag/AgCl reference electrode was placed in the recording chamber. Signals were amplified using a Multiclamp 700B amplifier (Molecular Devices), with a gain of 500 times. Raw data were recorded with a Bessel filter at 4 kHz and AC-coupled at 300 Hz. The analog voltage signals were digitized with a DigiData 1440A digitizing board (Molecular Devices) and acquired using pClamp 10 Clampex software (Molecular Devices).

### Light stimulation

The ambient light around the recording chamber measured with a light PAR meter (Model DP-355, Danoplus) was around 1.5 µmol m^−2^ s^−1^. White light was administered through a goose neck optical fiber, with a halogen lamp serving as the light source. To investigate the impact of various wavelengths of laser light on neural activity, we employed a multimode fiber-coupled laser source (89 North LDI), delivering red light at 635 nm, green light at 530 nm and blue light at 470 nm, all set at an intensity of 150 µmol m^−2^ s^−1^ for light stimulation. The light spot delivered through the optical fiber encompasses the entire arm. Laser activation was triggered by transistor–transistor logic (TTL) signals provided by an isolated pulse stimulator (Model 2100, A-M Systems), which was also utilized to deliver the same TTL pulse to the data acquisition board of the computer (DigiData 1440A digitizing board; Molecular Devices) to synchronize with electrophysiological recordings. To assess the relationship between light intensity and neural activity in the axial nerve cord, we first established a response curve by gradually increasing the intensity of different light wavelengths and determining the threshold of each wavelength needed to induce activity. The threshold was identified as the intensity level at which 50% of the light stimulation trials elicited a response. We subsequently compared the effects of light stimulation at 1×, 5× and 10× threshold intensity on neural activity. During the stimulation period, none of the light stimulation parameters altered the temperature of the recording chamber. To control for potential confounding effects of light order, a randomized presentation paradigm was employed. The sequence in which red, green and blue light stimuli were delivered was entirely random to prevent the influence of photoreceptor desensitization and adaptation periods induced by specific wavelengths. Prior to light exposure, baseline neural activity was recorded for 1 min. Subsequently, each light color was administered for a duration of 1 min, followed by a 1 min post-stimulation recording period to observe neural responses. A minimum of 30 s was allotted between light stimulations to allow for neural recovery. Data were collected from L1, L2, R1 and R2 arms of six different animals.

### Data analysis

Firing frequency and spike events were analyzed using Clampfit software (Molecular Devices). The Clampfit built-in threshold detection function was used to detect light stimulation-induced events. The threshold for event detection was set to 5 times the s.d. of the background noise. All statistical analyses were performed in GraphPad Prism (GraphPad Software 9.4.1). We used the D'Agostino-Pearson method to test the normality of the data. Data are presented as means±s.e.m. Statistical significance was evaluated using Student's *t*-test, and one-way ANOVA with Tukey's tests for multiple groups. Recorded potential signals were sorted using an unsupervised approach based on previously developed algorithms (Toosi et al., 2021) implemented in MATLAB R2022a (MathWorks, Natick, MA, USA). This algorithm involves band-pass filtering raw data within a frequency range of 300 to 3000 Hz to isolate neural activity. Spikes are detected by identifying signal peaks that exceed a threshold set at 5 times the s.d. of the noise. The time window for spikes spanned 2 ms, capturing 0.5 ms before and 1.5 ms after the detected spike point. Principal component analysis (PCA) was then applied to reduce the dimensionality of the spike waveforms, and a mixture of skew-*t* distributions model was used for clustering, with each cluster representing a different neuron.

## RESULTS

### Light induces neuronal activity in the axial nerve cord of the octopus arm

**Fig. 1.**
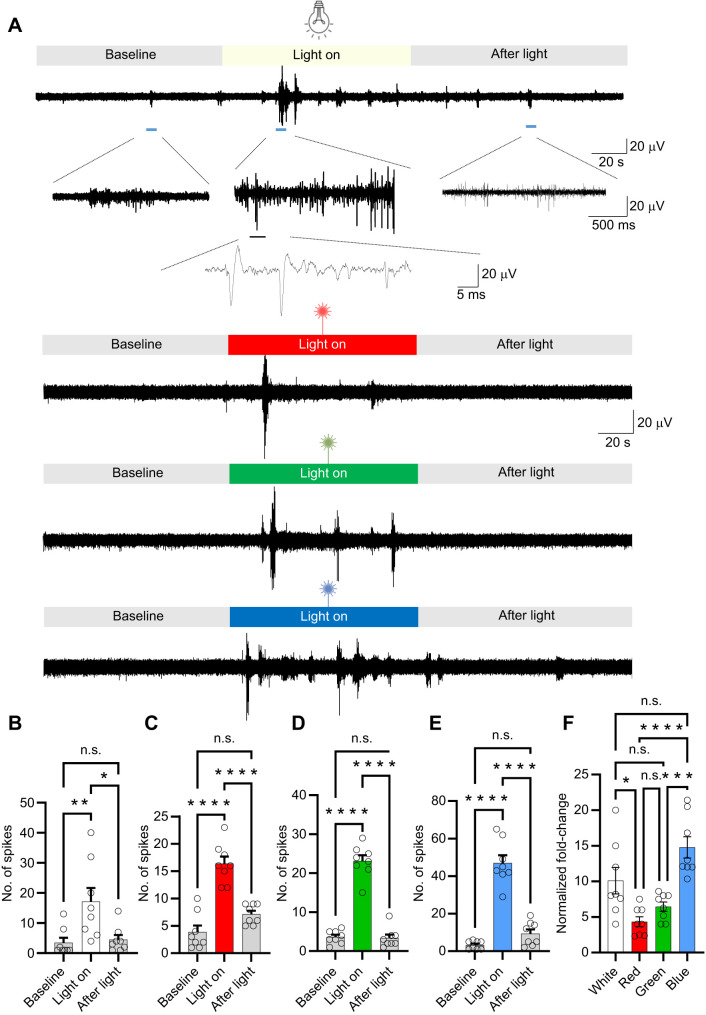
**Responses to light stimulation in the axial nerve cord of octopus arm: effect of wavelength on spike generation.** (A) Typical responses to light stimulation at different wavelengths, including white, red (635 nm), green (530 nm) and blue (470 nm) light. (B) The total number of spikes recorded in the axial nerve cord in response to white light (*N*=8 arms, 3 trials per arm) is significantly greater when compared with the control. However, after light stimulation, the spike level does not show a significant difference from the control. (C–E) Similarly, the total number of spikes recorded in response to red, green and blue light, respectively, is significantly greater than in the control. After light stimulation, the spike level does not differ significantly from the control. (F) Comparison of number of spikes induced by white, red, green and blue light, showing that blue light induces significantly more spikes. Data are means±s.e.m.; *P<0.05, **P<0.01, ***P<0.001, ****P<0.0001; one-way ANOVA with Tukey's *post hoc* tests.

To explore how light-induced activity is encoded within the arm, we euthanized the octopus and removed an arm, maintaining it physiologically and recording activity from the axial nerve cord. Initial experiments used white light stimulation, delivered via an optical fiber, with the emitted light covering the entire recording chamber. Recordings revealed spiking activity in response to white light stimulation (Fig. 1A). The shape of the multi-unit spikes recorded is shown in the lower panel of Fig. 1A. Statistical analysis confirmed that white light induced a significantly higher number of spikes in the axial nerve cord compared with the control baseline (Fig. 1B), demonstrating the sensitivity of the arm's neural circuits to local light stimulation.

### The strength and timing of the axial nerve cord response differ according to the wavelength of light stimuli

Red, green and blue light stimuli were used to assess responses to specific wavelengths of light in the axial nerve cord. Spiking activity increased in response to all wavelengths of light stimuli (Fig. 1A). An enlargement of the recording in lower panel of Fig. 1A shows the shape of the multi-unit spikes recorded. All tested wavelengths induced neural responses in the axial nerve cord, with all wavelengths evoking a significantly higher number of spikes compared with the control baseline (Fig. 1C–E). The results from light stimulation experiments are summarized in Table 1. Our findings indicate that blue light was the most effective wavelength in inducing neuronal activity, at least in the subset of neurons recorded. Blue light stimulation led to a significantly higher number of axial nerve cord spikes compared with its respective control, with a greater increase than that observed for green light (*P*=0.00072) or red light (*P*=0.00009) when compared with their respective controls; fold-change was as follows: white light 10.1±1.8, red light: 4.3±0.7, green light 6.4±0.7, blue light 14.8±1.5 (Fig. 1F).

**Table 1.** Neuronal spike rate during baseline, light stimulation and post-stimulation across different wavelengths

Light	Baseline (spikes min^−1^)	Light on (spikes min^−1^)	After light (spikes min^−1^)
White light	3.5±1.6	17.1±4.5	4.5±1.5
Red light	3.8±1.2	16.4±1.3	7.1±0.6
Green light	3.6±0.6	23.1±1.5	3.3±0.9
Blue light	3.2±0.7	47.1±4.1	9.4±2.3

Data are means±s.e.m.

**Fig. 2.**
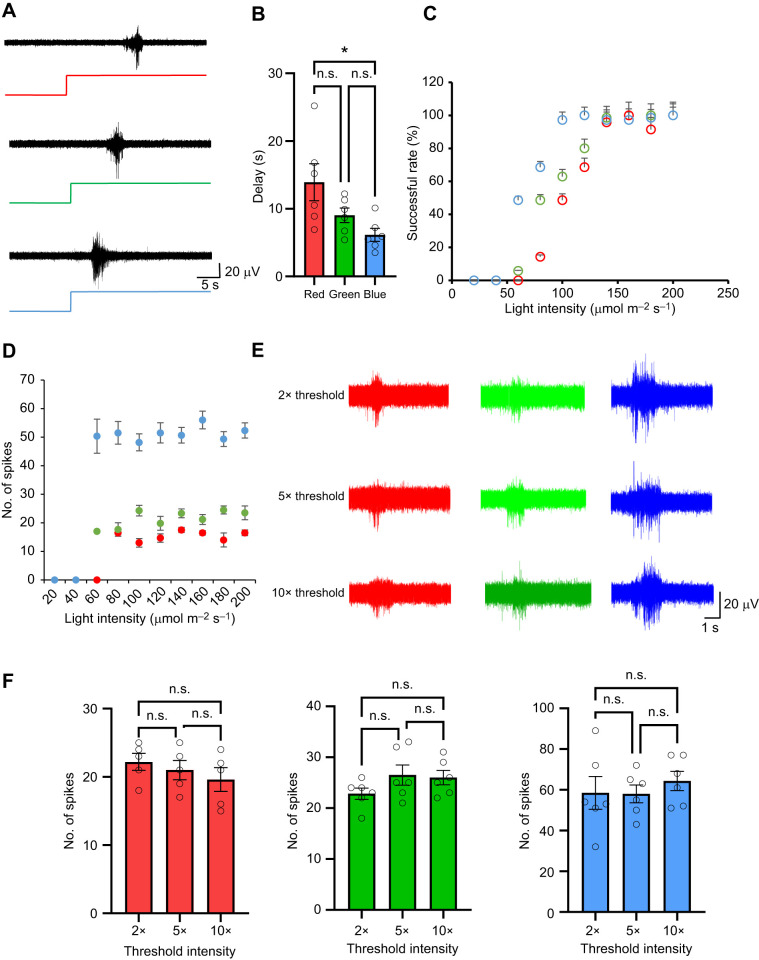
**Delay time and all-or-none feature of light-induced neuronal activity.** (A) A typical example of neuronal activity exhibits an obvious delay in response to light stimulation. (B) Statistical analysis demonstrates that blue light induces the shortest delay in neuronal activity, while red light induces the longest delay (*N*=6 arms, 3 trials per arm). (C) Response rates to different light stimulation intensities show that the threshold for blue light is around 60 µmol m^−2^ s^−1^, while the thresholds for green and red light are 80 and 100 µmol m^−2^ s^−1^, respectively. (D) Actual spike numbers as a function of light intensity for each wavelength. (E) Typical examples of neuronal activity induced by different wavelengths and intensities of light. (F) Summary data show that responses were induced by different intensities of red, green and blue light, with no significant differences observed within each light group. However, significant differences were observed when comparing responses between different light groups, as previously noted. Data are means±s.e.m.; *P<0.05; one-way ANOVA with Tukey's *post hoc* tests.

We observed a delay between light stimulation and the corresponding spike activity, as shown in Fig. 2A. The delay in spiking on axial nerve recordings was 6.1±0.8 s after blue light, 9±1.1 s after green light and 13.9±1.7 s after red light. The statistical analyses show that the delay time was significantly shorter when neurons were stimulated with blue light compared with red and green light (*P*<0.05; Fig. 2B). These findings indicate that the color of light has an impact on the timing of neuronal responses.

### Light intensity effects spiking in the axial nerve cord

We further investigated whether spiking in the axial nerve cord varied with different intensities of laser light. To accomplish this, we first established the stimulation threshold for different light wavelengths. We found that the response threshold for blue light was the lowest, approximately 60 µmol m^−2^ s^−1^, while red and green light stimulation had thresholds of around 80 and 100 µmol m^−2^ s^−1^ respectively (Fig. 2C). Spike numbers were analyzed as a function of light intensity for each wavelength. All wavelengths exhibited an all-or-none response, where spike numbers increased up to a certain light intensity threshold but showed no further change beyond this level (Fig. 2D). We evaluated the effect of different intensities (2, 5 and 10 times threshold) of each light wavelength on the spiking activity of the axial nerve cord (Fig. 2E). Increasing the light intensity past the threshold for a response increased the likelihood that spiking would occur but did not lead to an increase in spiking activity (Fig. 2F; red: *F*_2,15_=0.29, *P*=0.74; green: *F*_2,15_=0.63, *P*=0.54; blue: *F*_2,15_=0.39, *P*=0.68); there was no significant effect of changing light intensity on either number of spikes generated or latency to spiking in our preparation after the threshold intensity to elicit spiking was met. Our findings demonstrate a clear threshold-dependent response to light in the axial nerve cord.

### Aboral skin and oral skin and suckers are sites of light detection of the octopus arm

**Fig. 3.**
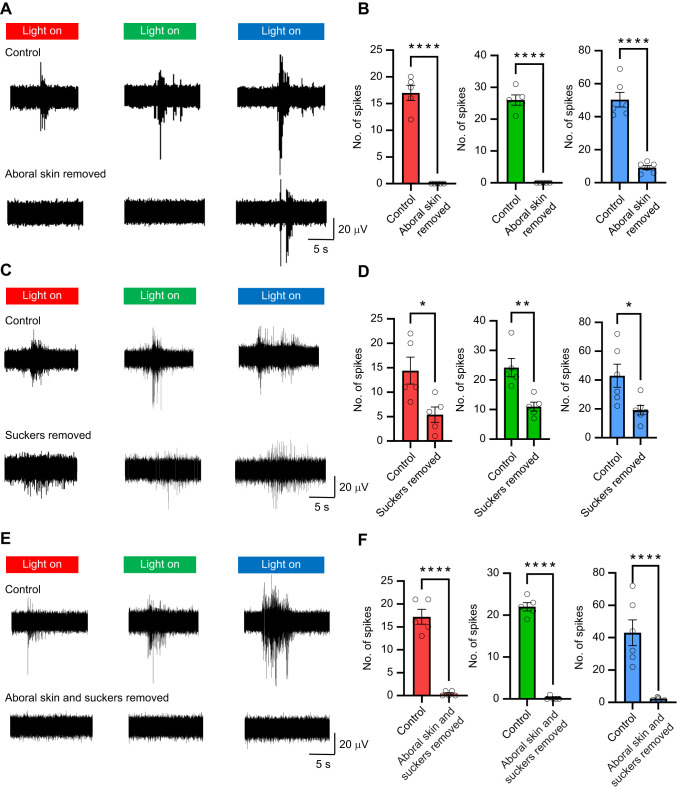
**Comparison of the effects of removal of different parts of the skin on light-induced activity.** (A) Typical activity recorded in the axial nerve cord in response to different light conditions in control and after removal of the aboral side of the skin. (B) Summary data show that spikes recorded in the axial nerve cord in response to red and green light stimulation are completely blocked after removal of the aboral skin. Removal of the aboral skin also significantly lowers the spike number induced by blue light compared with the control (*N*=5 arms, 3 trials per arm). (C) Typical activity recorded in the axial nerve cord in response to different light conditions in control and after removal of the suckers. (D) Summary results show that removal of the suckers does not block any light-induced responses, but significantly attenuates spike numbers in all lights tested (*N*=5 arms, 3 trials per arm). (E) Typical responses of the axial nerve cord during light stimulation, showing that all responses are blocked when aboral skin and suckers are removed. (F) Statistical results show that removal of aboral skin and suckers blocks neuronal activity induced by light (*N*=5 arms, 3 trials per arm). Data are means±s.e.m.; *P<0.05, **P<0.01, ****P<0.0001; paired Student's *t*-test.

To investigate the locations of light-sensing photoreceptors, we conducted two experiments, each designed to examine specific components of the arm. In the first experiment, we removed the aboral skin and compared the light-induced neuronal activity with that in the intact control. Removal of the aboral skin abolished responses to red and green light and attenuated responses to blue light (Fig. 3A). The summarized results of axial nerve cord activity in response to different wavelengths of light following aboral skin removal are shown in Table 2. Statistical analysis confirmed that neuronal responses to all tested wavelengths of light were significantly attenuated after removing the aboral skin (Fig. 3B), underscoring its critical role in extraocular light detection. In the second experiment, we removed all suckers and compared the light-induced neuronal responses with those of the intact control. Removal of the suckers slightly attenuated responses to red, green and blue light (Fig. 3C), with the summarized results presented in Table 3. Statistical analysis indicated that sucker removal significantly reduced neuronal responses to light (Fig. 3D), suggesting that the suckers have some light-sensing ability but play a lesser role in extraocular photoreception than the aboral skin. Importantly, when both the aboral skin and suckers were removed, no neuronal responses to light were detected (Fig. 3E,F), demonstrating that both structures contribute to light detection, with the aboral skin playing a dominant role.

**Table 2.** Neuronal responses to light after removal of aboral skin

Light	Control (spikes min^−1^)	Skin removal (spikes min^−1^)
Red light	17.1±1.9	N/A
Green light	23.9±1.6	N/A
Blue light	49.1±3.4	10.6±1.7

Data are means±s.e.m.

**Table 3.** Neuronal responses to light after removal of suckers

Light	Control (spikes min^−1^)	Sucker removal (spikes min^−1^)
Red light	14.4±3.3	4.1±1.5
Green light	24.3±2.1	9.2±1.7
Blue light	40.2±8.5	19.5±2.8

Data are means±s.e.m.

### There are distinct neuronal responses to red, green and blue light stimulation in the octopus arm

**Fig. 4.**
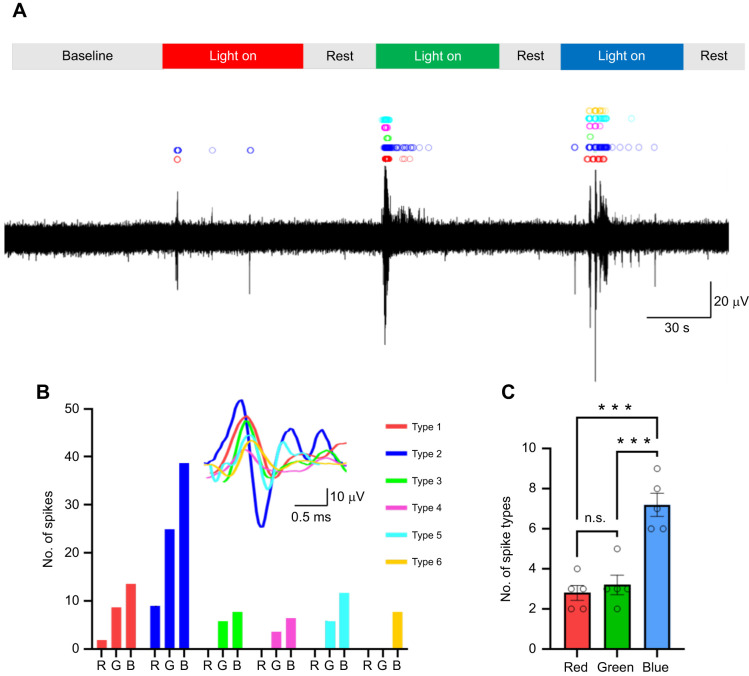
**Distinct neuronal responses induced by different wavelengths of light.** (A) An illustrative example demonstrates that spikes in response to red, green and blue light stimulation are distinct. (B) Summary data showing the distribution of spike types in the example in A. The inset shows overlapping averaged spike shapes detected from A. (C) Statistical analysis reveals a significant increase in the number of spike types firing in response to blue light (*N*=5 arms). Data are means±s.e.m.; ***P<0.001; one-way ANOVA with Tukey's *post hoc* tests.

A critical question in the understanding of extraocular photoreception is whether different neurons carry information about different wavelengths of light. Our electrophysiology recordings captured multiunit activity in response to red, green and blue light stimuli. We spike sorted this activity in response to different light stimulations to determine whether neurons respond to particular wavelengths of light. Our results indicate that neural activity can differ in response to wavelength (Fig. 4A). After performing spike sorting, we observed that red light stimulation elicited fewer distinct spike types, whereas blue light stimulation triggered a greater variety of spike firing patterns. Notably, certain neuronal spike types appeared exclusively during blue or green light stimulation. These findings suggest that different wavelengths of light evoke distinct neuronal spike profiles, reflecting wavelength-specific variation in neuronal activity. Fig. 4B summarizes the results in Fig. 4A, and Fig. 4C offers a representation of results from six animals.

### There is transmission of light-induced responses along the length of the octopus arm

**Fig. 5.**
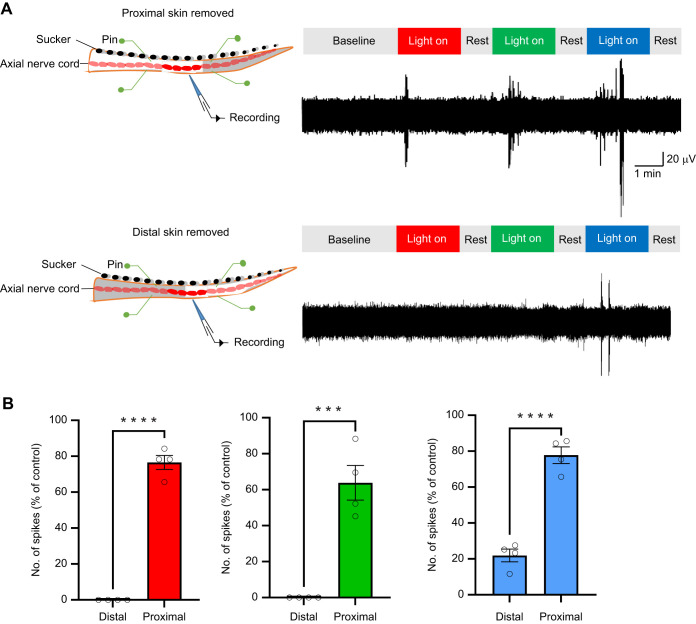
**Comparison of light-induced neuronal responses after removal of skin distal or proximal to the recording electrode.** (A) Typical examples of light-induced activity recorded in the axial nerve cord after removal of the distal (upper panel) and proximal (lower panel) skin. Octopus arms were secured to the Sylgard in the recording chamber by pins. Note that removal of the distal skin decreases the number of spikes in response to all light. (B) Summary results show that removal of the distal skin significantly lowers spike numbers in response to all light stimulation, while removal of the proximal skin does not significantly change spike numbers in response to light (*N*=4 arms, 3 trials per arm). Data are means±s.e.m.; ***P<0.001, ****P<0.0001; paired Student's *t*-test.

We also aimed to investigate propagation of signals within the octopus arm in response to light stimulation, focusing on the question of whether extraocular light reception results in transmission of signals toward the base of the arms and brain. To address this question, we recorded from the axial nerve cord after removing most of the skin on the arm, leaving a small region of skin intact, at either the distal or the proximal end (Fig. 5A).

We observed significant spiking in the axial nerve cord proximal to the region of skin photoreception, indicating signal propagation toward the base of the arm and brain. Likewise, recording distal to intact skin on the arm, with the rest of the aboral skin removed, also showed spiking, though less activity in our recordings. Additionally, with distal signal transmission, responses to red and green light were abolished and responses to blue light were significantly attenuated in our preparation (Fig. 5A,B). Under proximal signal transmission conditions, responses to all wavelengths of light were still observed but exhibited attenuation compared with control when the skin remained intact. We note that the suckers were intact in this preparation, but our previous experiments (Fig. 3C,D) showed that their contribution to light-induced responses was small. These findings provide evidence for proximal transmission of light-induced signals in the octopus arm, as phototransduction-generated activity in the distal skin appears to propagate toward the central nervous system.

## DISCUSSION

In this study, we show for the first time that local light stimulation induces neural responses in the axial nerve cord of octopus arms. The axial nerve cord serves as a critical conduit for transmitting sensory information to the central brain and other arms. Our findings suggest that light-induced signals detected in the periphery, such as in the skin or suckers, are processed in the axial nerve cord and may be transmitted to central neural regions, based on the anatomical connections of the axial nerve cord.

Blue, green and red wavelengths all induce neural responses in the axial nerve cord. Our data indicate that blue light is the most potent inducer of neural activity in the octopus arm, consistent with previous findings that LACE behavior is maximally sensitive to blue light (Ramirez and Oakley, 2015). Spectral analysis from the eyes of another species of octopus (*O. vulgaris*) also shows maximal sensitivity to 474 nm light (Brown and Brown, 1958). The strong response to blue light and the LACE response highlight the potential role of blue light sensitivity in aiding octopus behavior in environments where blue light predominates.

We note that the neural activity that we recorded in response to light stimulation exhibited significant delay relative to stimulus onset. For example, the delay in response to blue light stimulation was around 6 s. This is consistent with the LACE response; Ramirez and Oakley (2015) observed a LACE delay with maximum expansion chromatophore latency in response to blue light in about 10 s. Katz et al. (2021) reported a shorter delay in the behavioral response to local arm light stimulation in intact adult octopus, with arm retraction occurring in just 0.77 s. The differences in delay times across these studies may be related to variation in experimental preparations. Our study used isolated whole-arm preparations, Katz et al. (2021) used whole animals, and Ramirez and Oakley (2015) used dissected funnels. It may also be that the nerve fibers of the axial nerve cord that we recorded from are associated with the LACE response but would not be driving the immediate response to light stimulation seen with negative phototaxis.

The delayed timing of the neural response was surprising in relation to known opsin responses. Typically, the depolarization response resulting from opsin activation ranges from a few milliseconds to tens of milliseconds (Lamb and Pugh, 1992). It is possible that the activity recorded from the axial nerve cord in our study might not directly reflect opsin activation but rather relay of activity from chromatophores. Our preparation was an isolated arm and chromatophores are known to be able to change conformation in response to light stimulation without visual input (e.g. Florey, 1966). Additionally, the activation of reflectin protein phosphorylation by acetylcholine can alter skin color (Levenson et al., 2016), and certain fish species can change skin color without visual input (Zhang et al., 2024). These findings suggest that the skin itself has signaling pathways that control pigment cells, although the exact mechanisms require further investigation.

Another explanation for the delayed onset of neural activity to extraocular light stimulation is that opsin and related signaling pathways may exist in different functional states. In whole animals, the central nervous system (CNS) might provide tonic signals that enhance pathway sensitivity, leading to faster responses. In isolated arms, these pathways may be in a more quiescent state, requiring more time for signal accumulation to reach activation thresholds. This suggests a dynamic interaction between CNS modulation and local signaling, warranting further investigation.

This study diverges from previous findings in that, in our experiments, the neuronal activity in octopus arms exhibits an all-or-none response to light stimulation. It is unclear how this response is mediated or how it relates to behavior. If opsin or chromatophores are specialized to respond only up to a certain intensity, the neural response could peak at or slightly above this threshold, regardless of further light intensity increases. While the response of opsin to light is typically graded (Palczewski, 2006), some opsins have evolved to specialize in detecting specific characteristics of light, such as color or polarization. For instance, RH1 pigments in deep-sea creatures are optimized for detecting downwelling sunlight at approximately 480 nm (Yokoyama, 2008), while specialized opsins in insects (McCulloch et al., 2022) and crustaceans (Roberts et al., 2011) enable detection of polarized light. These observations suggest that the all-or-none neural response in octopus arms may represent a unique adaptation, potentially reflecting the specialization of peripheral light-detecting systems to specific environmental or behavioral contexts, warranting further investigation into their functional significance.

Our results demonstrate that light-induced responses in the axial nerve cord of octopus arms exhibit an all-or-none pattern, where neural activity occurs only after a threshold intensity of light is reached. This response pattern contrasts with previous findings in *O. vulgaris* and *S. officinalis*, which showed sensitivity to varying brightness levels (Messenger et al., 1973; Buresch et al., 2015). While intensity sensitivity plays a role in light detection across cephalopods, our results suggest that extraocular photoreceptive responses in the octopus arm are tuned to detect specific light thresholds rather than respond proportionally to brightness changes. The consistent threshold intensities required to elicit neural responses across different wavelengths in our experiments support the interpretation that the observed responses are driven by specific wavelengths and threshold intensity, rather than brightness alone. These findings highlight the need for further exploration of the mechanisms underlying the all-or-none responses observed in octopus arms, as well as their functional relationship with chromatophore behavior and environmental adaptations.

Our study demonstrates that light-induced neuronal responses in octopus arms depend primarily on the aboral skin. Removal of the aboral skin completely abolished responses to red and green light and significantly reduced responses to blue light, indicating its central role in light sensing. In contrast, removing the suckers attenuated, but did not eliminate, neuronal responses, suggesting that while the suckers and surrounding skin contribute to light detection, their role is less significant compared with the aboral skin. We presume that extraocular reception on the oral (sucker-bearing) side of the arm is generated through the skin of the sucker and the area of the arm directly surrounding the suckers. While questions remain about light sensing on the oral side of the arm, these results support the hypothesis that the primary light-sensing structures reside in the aboral skin, where chromatophores are most densely distributed. This suggests a possible functional relationship between the location of extraocular photosensation and the coordination of chromatophore activity, such as LACE, or as noted above, that our recordings are showing secondary activity related to the LACE response, rather than a response to opsin stimulation directly.

Spike-sorting analyses revealed that distinct wavelengths of light elicit varied neuronal activity, as evidenced by differences in spike profiles and the number of spike types detected. Blue light stimulation activated a broader diversity of neuron types compared with red or green light, suggesting it recruits a larger population of responsive neurons. This variation may result from differential sensitivity thresholds or spectral tuning properties of opsin molecules within the photoreceptive tissues of the arm (Ramirez and Oakley, 2015). Although cephalopods are thought to possess a single type of visual pigment (Brown and Brown, 1958), the observed differences in neural responses suggest downstream processing mechanisms, such as modulation by cellular structures, post-translational modification of opsin or localized differences in photoreceptor distribution. Additionally, RNA editing and alternative splicing (Liscovitch-Brauer et al., 2017) could produce functionally distinct opsin isoforms, fine-tuning responses to specific wavelengths. For instance, a single amino acid variation in cuttlefish ventral skin opsin compared with its retinal counterpart may alter spectral sensitivity (Mäthger et al., 2010). These findings highlight the complexity of wavelength-specific neuronal activity, shaped by molecular, cellular and circuit-level mechanisms in the octopus arm.

Removal of the distal end of the skin significantly attenuated light-induced neuronal responses recorded in the axial nerve cord, while proximal skin removal had minimal effect. With the electrode positioned in the middle of the arm, our results indicate that ascending signaling predominates over descending signaling, at least from the area of the cerebrobrachial tract from which we were recording. These findings highlight the role of the distal regions of the arm in initiating light-sensitive signals, which appear to propagate proximally along the axial nerve cord toward the central nervous system, providing environmental sensory input. While our results suggest a stronger light-induced response in the distal arm regions, the light stimulation in this study covered the entire arm, and future experiments will need to target light specifically to the distal, middle and proximal regions while adjusting electrode positions accordingly to better determine the distribution of ascending extraocular receptors and the functional relevance of this signaling. Behavioral studies have shown stronger light-induced responses at the arm tips (Katz et al., 2021), aligning with our results, though these comparisons should be interpreted cautiously given differences in experimental approaches and recording conditions. This does not conflict with the findings of Al-Soudy et al. (2021), which focused on the suckers. Al-Soudy et al. (2021) demonstrated that Ov-GRK1, a light-sensing molecule, is expressed in the sucker rims and suggested a potential light-sensing capability in these structures. This aligns with our findings, as the photosensitive suckers and surrounding skin may contribute to the light-induced neuronal responses we recorded in the axial nerve cord, particularly from distal regions of the arm.

The recorded neuronal activity in the axial nerve cord is consistent with local signaling associated with LACE mechanisms, specifically mediated by dermal photoreceptors and local sensory circuits in the octopus arm. However, it is also possible that this activity represents sensory input or intermediate processing that feeds into motor systems, rather than exclusively reflecting LACE. The evidence of ascending transmission within the axial nerve cord supports the idea that both localized responses, such as chromatophore expansion, and broader sensory-motor pathways may contribute to the light-induced signals we recorded. Although the isolated arm preparation used in this study eliminates brain input, and central coordination suggests a primary role for local processing, the presence of ascending signals highlights the potential for these sensory inputs to influence central neural circuits in intact animals and inform broader behavioral responses.

These findings provide evidence that light-induced neural activity in the octopus arm is mediated through the skin and transmitted via the axial nerve cord, supporting the role of extraocular photoreception as a peripheral sensory mechanism. The heightened sensitivity to blue light aligns with prior studies on chromatophore expansion and may suggest a functional adaptation to the blue-light-dominated environments in which octopuses often reside. While our study provides electrophysiological evidence of light-induced neural activity, the biological significance of these responses remains to be fully understood. It is possible that such extraocular sensory input contributes to behaviors such as camouflage by providing localized sensory feedback to the central nervous system for integration. Additionally, the observed ascending transmission of light-induced signals highlights the potential for peripheral photoreception to inform central neural processes and influence sensory-motor coordination. These results lay the groundwork for future studies to explore the functional relevance of extraocular photoreception in octopus behavior and ecology.
